# Assume, Guarantee or Repair

**DOI:** 10.1007/978-3-030-45190-5_12

**Published:** 2020-03-13

**Authors:** Hadar Frenkel, Orna Grumberg, Corina Pasareanu, Sarai Sheinvald

**Affiliations:** 8 Johannes Kepler University, Linz, Austria; 9 University of Birmingham, Birmingham, UK; 10 Department of Computer Science, The Technion, Haifa, Israel; 11 Carnegie Mellon University and NASA Ames Research Center, CA Mountain View, USA; 12 Department of Software Engineering, Braude College of Engineering, Karmiel, Israel

## Abstract

We present Assume-Guarantee-Repair (AGR) – a novel framework which not only verifies that a program satisfies a set of properties, but also *repairs* the program in case the verification fails. We consider *communicating programs* – these are simple C-like programs, extended with synchronous communication actions over communication channels. Our method, which consists of a learning-based approach to assume-guarantee reasoning, performs verification and repair simultaneously: in every iteration, AGR either makes another step towards proving that the (current) system satisfies the specification, or alters the system in a way that brings it closer to satisfying the specification. We manage handling infinite-state systems by using a finite abstract representation, and reduce the semantic problems in hand – satisfying complex specifications that also contain first-order constraints – to syntactic ones, namely membership and equivalence queries for regular languages. We implemented our algorithm and evaluated it on various examples. Our experiments present compact proofs of correctness and quick repairs.

## 1 Introduction

Verification of large-scale systems is a main challenge in the field of formal verification. Often, the verification process of such a system does not scale well. *Compositional verification* aims to verify small components of a system separately, and from the correctness of the individual components, to conclude the correctness of the entire system. This, however, is not always possible, since the correctness of a component often depends on the behavior of its environment.

The Assume-Guarantee (AG) style compositional verification [22,26] suggests a solution to this problem. The simplest AG rule checks if a system composed of components $M_1$ and $M_2$ satisfies a property $P$ by checking that $M_1$ under assumption $A$ satisfies $P$ and that any system containing $M_2$ as a component satisfies $A$. Several frameworks have been proposed to support this style of reasoning. Finding a suitable assumption $A$ is then a common challenge in such frameworks.

In this work, we present *Assume-Guarantee-Repair* (AGR) – a fully automated framework which applies the Assume-Guarantee rule, and while seeking a suitable assumption $A$, incrementally repairs the given program in case the verification fails. Our framework is inspired by [24], which presented a learning-based method to finding an assumption $A$, using the $L^*$ [5] algorithm for learning regular languages.

Our AGR framework handles *communicating programs*. These are infinite-state C-like programs, extended with the ability to synchronously read and write messages over communication channels. We model such programs as finite-state automata over an *action alphabet*, which reflects the program statements. The accepting states in these automata model points of interest in the program that the specification can relate to. The automata representation is similar in nature to that of control-flow graphs. Its advantage, however, is in the ability to exploit an automata-learning algorithm such as $L^*$.

* This research was partially supported by the Technion Hiroshi Fujiwara Cyber Security Research Center, the Israel National Cyber Directorate and the Israel Science Foundation (ISF)

The composition of the two program components, $M_1$ and $M_2$, denoted $M_1||M_2$, synchronizes on read-write actions on the same channel. Between two synchronized actions, the individual actions of both systems interleave.

```
while(true)
    password:=readInput;
    while(password≤ 999)
        password:=readInput;
    password2:=encrypt(password);
```

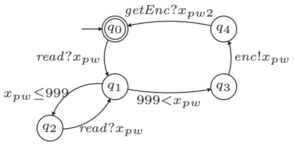

Fig. 1: Modeling a communicating program as an automaton $M_2$

Figure 1 presents the code of a communicating program (left) and its corresponding automaton $M_2$ (right). The automaton alphabet consists of constraints (e.g. $x_{pw} \leq 999$), assignment actions (e.g. $y_{pw} := 2 \cdot y_{pw}$ in $M_1$ of Figure 2), and communication actions (e.g. $enc!x_{pw}$ sends the value of variable $x_{pw}$ over channel $enc$, and $getEnc?x_{pw2}$ reads a value to $x_{pw2}$ on channel $getEnc$).

The specification $P$ is modeled as an automaton that does not contain assignment actions. It may contain communication actions in order to specify behavioral requirements, as well as constraints over the variables of both system components, that express requirements on their values in various points in the runs.

Consider, for example, the program $M_1$ and the specification $P$ seen in Figure 2, and the program $M_2$ of Figure 1. $M_2$ reads a password on channel $read$ to the variable $x_{pw}$, and once it is long enough (has at least four digits), it sends the value of $x_{pw}$ to $M_1$ through channel $enc$. $M_1$ reads this value to variable $y_{pw}$ and then applies a simple function that changes its value, and sends the changed variable back to $M_2$. The property $P$ reasons about the parallel run of the two programs. The pair $(getEnc?x_{pw2}, getEnc!y_{pw})$ denotes a synchronization of $M_1$ and $M_2$ on channel $getEnc$. $P$ makes sure that the parallel run of $M_1$ and $M_2$ always reads a value and then encrypts it – a temporal requirement. In addition, it makes sure that the value after encryption is different than the original value, and that there is no overflow – both are semantic requirements on the program variables. That is, $P$ expresses temporal requirements that contain first order constraints. In case one of the requirements does not hold, $P$ reaches the state $r_4$ which is an error state. Note that $P$ here is not complete, for simplicity of presentation (see Definition 5 for a formal definition of a complete program).

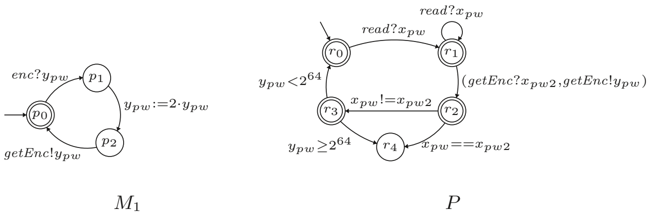

Fig. 2: The programs $M_1$, $M_2$, and the specification $P$

The $L^*$ algorithm aims at learning a regular language $U$. Its entities consist of a *teacher* – an oracle who answers *membership queries* ("is the word $w$ in $U$?") and *equivalence queries* ("is $\mathcal{A}$ an automaton whose language is $U$?"), and a *learner*, who iteratively constructs a finite deterministic automaton $\mathcal{A}$ for $U$ by submitting a sequence of membership and equivalence queries to the teacher.

In using the $L^*$ algorithm for learning an assumption $A$ for the AG-rule, membership queries are answered according to the satisfaction of the specification $P$: If $M_1||t$ satisfies $P$, then the trace $t$ in hand should be in $A$. Otherwise, $t$ should not be in $A$. Once the learner constructs a stable system $A$, it submits an equivalence query. The teacher then checks whether $A$ is a suitable assumption, that is, whether $M_1||A$ satisfies $P$, and whether the language of $M_2$ is contained in the language of $A$. According to the results, the process either continues or halts with an answer to the verification problem. The learning procedure aims at learning the weakest assumption $A_w$, which contains all the traces that in parallel with $M_1$ satisfy $P$ . The key observation that guarantees termination in [24] is that the components in this procedure – $M_1$,$M_2$, $P$ and $A_w$ – are all regular.

Our setting is more complicated, since the traces in the components – both the programs and the specification – contain constraints, which are to be checked semantically and not syntactically. These constraints may cause some traces to become infeasible. For example, if a trace contains an assignment $x := 3$ followed by a constraint $x \geq 4$ (modeling an "if" statement), then this trace does not contribute any concrete runs, and therefore does not affect the system behavior. Thus, we must add feasibility checks to the process.

Constraints in the specification also pose a difficulty, as satisfiability of a specification is determined by the semantics of the constraints and not only by the language syntax, and so there is more here to check than standard language containment. Moreover, in our setting $A_w$ above may no longer be regular, see Example 3. However, our method manages to overcome this problem.

As we have described above, not only do we construct a learning-based method for the AG-rule for communicating programs, but we also repair the programs in case the verification fails. An AG-rule can either conclude that $M_1||M_2$ satisfies $P$, or return a real, non-spurious counterexample of a computation $t$ of $M_1||M_2$ that violates $P$. In our case, instead of returning $t$, we repair $M_2$ in a way that eliminates this counterexample. Our repair is both syntactic and semantic, where for semantic repair we use *abduction* [25] to infer a new constraint which makes the counterexample $t$ infeasible.

Consider again $M_1$ and $P$ of Figure 2 and $M_2$ of Figure 1. The composition $M_1||M_2$ does not satisfy $P$. For example, if the initial value of $x_{pw}$ is $2^{63}$, then after encryption the value of $y_{pw}$ is $2^{64}$, violating $P$. Our algorithm finds a bad trace during the AG stage which captures this bad behavior, and the abduction in the repair stage finds a constraint that eliminates it: $x_{pw} < 2^{63}$, and inserts this constraint to $M_2$.

Following this step we now have an updated $M_2$, and we continue with applying the AG-rule again, using information we have gathered in the previous steps. In addition to removing the error trace, we update the alphabet of $M_2$ with the new constraint.

Continuing our example, in a following iteration AGR will verify that the repaired $M_2$ together with $M_1$ satisfy $P$, and terminate.

Thus, AGR operates in a verify-repair loop, where each iteration runs a learning-based process to determine whether the (current) system satisfies $P$, and if not, eliminates bad behaviors from $M_2$ while enriching the set of constraints derived from these bad behaviors, which often leads to a quicker convergence. In case the current system does satisfy $P$, we return the repaired $M_2$ together with an assumption $A$ that abstracts $M_2$ and acts as a smaller proof for the correctness of the system.

We have implemented a tool for AGR and evaluated it on examples of various sizes and of various types of errors. Our experiments show that for most examples, AGR converges and finds a repair after 2-5 iterations of verify-repair. Moreover, our tool generates assumptions that are significantly smaller then the (possibly repaired) $M_2$, thus constructing a compact and efficient proof of correctness.

**Contributions** To summarize, the main contributions of this paper are:

1. A learning-based Assume-Guarantee algorithm for infinite-state communicating programs, which manages to overcome the difficulties such programs present. In particular, our algorithm overcomes the inherent irregularity of the first-order constraints in these programs, and offers syntactic solutions to the semantic problems they impose.
2. An Assume-Guarantee-Repair algorithm, in which the Assume-Guarantee and the Repair procedures intertwine to produce a repaired program which, due to our construction, maintains many of the "good" behaviors of the original program. Moreover, in case the original program satisfies the property, our algorithm is guaranteed to terminate and return this conclusion.
3. An incremental learning algorithm that uses query results from previous iterations in learning a new language with a richer alphabet.
4. A novel use of abduction to repair communicating programs over first order constraints.
5. An implementation of our algorithm, demonstrating the effectiveness of our framework.

**Related Work** Assume-guarantee style compositional verification [22,26] has been extensively studied. The assumptions necessary for compositional verification were first produced manually, limiting the practicality of the method.

More recent works [9,16,14,6] proposed techniques for automatic assumption generation using learning and abstraction refinement techniques, making assume-guarantee verification more appealing. In [24,6] alphabet refinement has been suggested as an optimization, to reduce the alphabet of the generated assumptions, and consequently their sizes. This optimization can easily be incorporated into our framework as well.

Other learning-based approaches for automating assumption generation have been described in [7,17,8]. All these works address non-circular rules and are limited to finite state systems. Automatic assumption generation for circular rules is presented in [12,13], using compositional rules similar to the ones studied in [21,23].

Our approach is based on a non-circular rule but it targets complex, infinite-state concurrent systems, and addresses not only verification but also repair. The compositional framework presented in [19] addresses $L^*$-based compositional verification and synthesis but it only targets finite state systems.

Also related is the work in [18], which addresses automatic synthesis of circular compositional proofs based on logical abduction; however the focus of that work is sequential programs, while here we target concurrent programs. A sequential setting is also considered in [3], where abduction is used for automatically generating a program environment. Our computation of abduction is similar to that of [3]. However, we require our constraints to be over a predefined set of variables, while they look for a minimal set.

The approach presented in [27] aims to compute the *interface* of an infinite-state component. Similar to our work, the approach works with both over- and under- approximations but it only analyzes one component at a time. Furthermore, the component is restricted to be deterministic (necessary for the permissiveness check). In contrast we use both components of a system to compute the necessary assumptions, and as a result they can be much smaller than in [27]. Furthermore, we do not restrict the components to be deterministic and, more importantly, we also address the system repair in case of dissatisfaction.

## 2 Communicating Programs

In this section we present the notion of *communicating programs*. These are C-like programs, extended with the ability to synchronously read and write messages over communication channels. We model such programs as automata over an *action alphabet* that reflects the program statements. The alphabet includes *constraints*, which are quantifier-free first-order formulas, representing the conditions in *if* and *while* statements. It also includes *assignment statements* and $read$ and $write$ *communication actions*. The automata representation is similar in nature to that of a control-flow graph. Its advantage, however, is in the ability to exploit an automata-learning algorithm such as $L^*$ for its verification.

We first formally define the alphabet over which communicating programs are defined. Let $G$ be a finite set of communication channels. Let $X$ be a finite set of variables (whose ordered vector is $\bar{x}$) and $D$ be a (possibly infinite) data domain. For simplicity, we assume that all variables are defined over $D$. The elements of $D$ are also used as constants in arithmetic expressions and constraints.

**Definition 1.** *An* action alphabet *is* $\alpha = \mathcal{G} \cup \mathcal{E} \cup \mathcal{C}$ *where:*

1. $\mathcal{G} \subseteq \{\, g?x_1, g!x_1, (g?x_1, g!x_2), (g!x_1, g?x_2) \mid g \in G, x_1, x_2 \in X\}$ *is a finite set of* communication actions. $g?x$ *is a* read *action of a value to the variable $x$ through channel $g$, and $g!x$ is a* write *action of the value of $x$ on channel $g$. We use $g * x$ to indicate some action, either* read *or* write*, through $g$. The pairs $(g?x_1, g!x_2)$ and $(g!x_1, g?x_2)$ represent a synchronization of two programs on read-write actions over channel $g$ (defined later).*
2. $\mathcal{E} \subseteq \{\, x := e \mid e \in E, x \in X\}$ *is a finite set of* assignment statements*, where $E$ is a set of expressions over $X \cup D$.*
3. *$\mathcal{C}$ is a finite set of constraints over $X \cup D$.*

**Definition 2.** *A* communicating program *(or, a program) is* $M = \langle Q, X, \alpha, \delta, q_0, F\rangle$*, where:*

1. *$Q$ is a finite set of states and $q_0 \in Q$ is the initial state.*
2. *$X$ is a finite set of variables that range over $D$.*
3. *$\alpha = \mathcal{G} \cup \mathcal{E} \cup \mathcal{C}$ is the action alphabet of $M$.*
4. *$\delta \subseteq Q \times \alpha \times Q$ is the transition relation, where for each $q \in Q$, only one of the following holds:*
   - *$\alpha \in \mathcal{C}$ for all $(q, \alpha, q') \in \delta$*
   - *$\alpha \in \mathcal{G} \cup \mathcal{E}$ for all $(q, \alpha, q') \in \delta$*

*That is, for each state it holds that either all outgoing edges are labeled with constraints, or that all outgoing edges are labeled with assignments or communication actions.*
5. *$F \subseteq Q$ is the set of accepting states.*

The words that are read along a communicating program are a *symbolic representation* of the program behaviors. We refer to such a word as a *trace*. Each such trace induces *concrete runs* of the program, which are formed by concrete assignments to the program variables in a way that conforms with the actions along the word.

We now formally define these notions.

**Definition 3.** *A* path *in a program $M$ is a finite sequence of states and actions $p = (q_0, a_1, q_1, \ldots, a_n, q_n)$, starting with the initial state $q_0$, such that $\forall 0 \le i < n$ we have $(q_i, a_{i+1}, q_{i+1}) \in \delta$. The* induced trace *of $p$ is the sequence $t = (a_1, \ldots, a_n)$ of the actions in $p$. If $q_n$ is accepting, then $t$ is an* accepted trace *of $M$.*

From now on we assume that every trace we discuss is induced by some path. We turn to define the concrete runs of the program.

**Definition 4.** *Let $t = (a_1, \ldots, a_n)$ be a trace and let $(\beta_0, \ldots, \beta_n)$ be a sequence of valuations (i.e., assignments to the program variables)*[4]*. Then a sequence $r = (\beta_0, a_1, \beta_1, a_2, \ldots, a_n, \beta_n)$ is a* run *of $t$ if the following holds.*

---
[4] Such valuations are usually referred to as states. We do not use this terminology here in order not to confuse them with the states of the automaton.

1. *$\beta_0$ is an arbitrary valuation.*
2. *If $a_i = g?x$, then $\beta_i(y) = \beta_{i-1}(y)$ for every $y \neq x$. Intuitively, $x$ is arbitrarily assigned by the read action, and the rest of the variables are unchanged.*
3. *If $a_i$ is an assignment $x := e$, then $\beta_i(x) = e[\bar{x} \leftarrow \beta_{i-1}(\bar{x})]$ and $\beta_i(y) = \beta_{i-1}(y)$ for every $y \neq x$.*
4. *If $a_i = (g?x, g!y)$ or $a_i = (g!y, g?x)$ then $\beta_i(x) = \beta_{i-1}(y)$ and $\beta_i(z) = \beta_{i-1}(z)$ for every $z \neq x$. That is, the effect of a synchronous communication on a channel is that of an assignment.*
5. *If $a_i$ does not involve a read or an assignment, then $\beta_i = \beta_{i-1}$.*
6. *Finally, if $a_i$ is a constraint in $\mathcal{C}$, then $\beta_i(\bar{x}) \models a_i$ (and since $a_i$ does not change the variable assignments, then $\beta_{i-1}(\bar{x}) \models a_i$ holds as well).*

*We say that t is* feasible *if there exists a run of t.*

The *symbolic language* of $M$, denoted $\mathcal{T}(M)$, is the set of all *accepted* traces induced by paths of $M$. The *concrete language* of $M$ is the set of all runs of accepted traces in $\mathcal{T}(M)$. We will mostly be interested in feasible traces, which represent (concrete) runs of the program. Intuitively, the symbolic language of a program $M$ corresponds to its syntactic behavior, while the concrete language corresponds to the semantics of the program.

*Example 1.*
- The trace $(x := 2 \cdot y,\ g?x,\ y := y+1,\ g!y)$ is feasible, as it has a run $(x = 1, y = 3), (x = 6, y = 3), (x = 20, y = 3), (x = 20, y = 4), (x = 20, y = 4)$.
- The trace $(g?x,\ x := x^2\ , x < 0)$ is not feasible since no $\beta$ can satisfy the constraint $x < 0$ if $x := x^2$ is executed beforehand.

### 2.1 Parallel Composition

We now describe and define the parallel run of two communicating programs, and the way in which they communicate.

Let $M_1$ and $M_2$ be two programs, where $M_i = \langle Q_i, X_i, \alpha_i, \delta_i, {q_0}^i, F_i \rangle$ for $i \in \{1, 2\}$. Let $G_1, G_2$ be the sets of communication channels occurring in actions of $M_1, M_2$, respectively. We assume $X_1 \cap X_2 = \emptyset$.

The *interface alphabet* $\alpha I$ of $M_1$ and $M_2$ consists of all communication actions on channels that are common to both components. That is, $\alpha I = \{\, g?x,\ g!x \mid g \in G_1 \cap G_2,\ x \in X_1 \cup X_2 \}$.

In *parallel composition*, the two components synchronize on their communication interface only when one component writes data through a channel, and the other reads it through the same channel. The two components cannot synchronize if both are trying to read or both are trying to write. We distinguish between communication of the two components with each other (on their common channels), and their communication with their environment. In the former case, the components must "wait" for each other in order to progress together. In the latter case, the communication actions of the two components interleave asynchronously.

Formally, the *parallel composition* of $M_1$ and $M_2$, denoted $M_1||M_2$, is the program $M = \langle Q, x, \alpha, \delta, q_0, F \rangle$ defined as follows.

1. $Q = (Q_1 \times Q_2) \cup (Q_1' \times Q_2')$, where $Q_1'$ and $Q_2'$ are new copies of $Q_1$ and $Q_2$, respectively. The initial state is $q_0 = (q_0^1, q_0^2)$.
2. $X = X_1 \cup X_2$.
3. $\alpha = \{\, (g?x_1, g!x_2), (g!x_1, g?x_2) \mid g{*}x_1 \in (\alpha_1 \cap \alpha I)$ and $g{*}x_2 \in (\alpha_2 \cap \alpha I)\} \cup ((\alpha_1 \cup \alpha_2) \setminus \alpha I)$. That is, the alphabet includes pairs of read-write communication actions on channels common to $M_1$ and $M_2$. It also includes individual actions of $M_1$ and $M_2$ – assignment actions, constraints and communication actions which are not communications on common channels.

4. $\delta$ is defined as follows.
   (a) For $(g * x_1, g * x_2) \in \alpha$:
      i. $\delta((q_1, q_2), (g * x_1, g * x_2)) = (q_1', q_2')$.
      ii. $\delta((q_1', q_2'), x_1 == x_2) = (\delta_1(q_1, g * x_1), \delta_2(q_2, g * x_2))$.
   That is, when a communication is performed synchronously in both components, the data is transformed through the channel from the writing component to the reading component. As a result, the values of $x_1$ and $x_2$ equalize. This is enforced in $M$ by adding a transition labeled by the constraint $x_1 == x_2$ that immediately follows the synchronous communication.
   (b) For $a \in \alpha_1 \setminus \alpha I$ we define $\delta((q_1, q_2), a) = (\delta_1(q_1, a), q_2)$.
   (c) For $a \in \alpha_2 \setminus \alpha I$ we define $\delta((q_1, q_2), a) = (q_1, \delta_2(q_2, a))$.
   That is, on actions that are not in the interface alphabet, the two components interleave.
5. $F = F_1 \times F_2$

Figure 3 demonstrates the parallel composition of components $M_1$ and $M_2$ of Figures 1 and 2. The program $M = M_1 || M_2$ reads a password from the environment through channel $read$. The two components synchronize on channels $enc$ and $getEnc$.

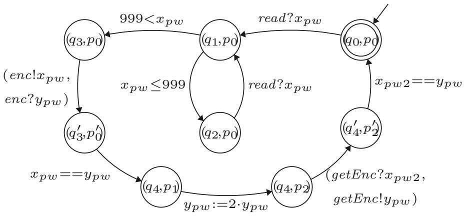

Fig. 3: Parallel composition $M = M_1 || M_2$ of components $M_1$ and $M_2$ from Figures 1, 2

## 3 Regular Properties and Their Satisfaction

In this section we define the syntax and semantics of the properties that we consider. These are properties that can be represented as finite automata, hence the name *regular*. However, the alphabet of such automata includes communication actions and first-order constraints over program variables. Thus, such automata are suitable for specifying the desired and undesired behaviors of communicating programs over time.

In order to define our properties, we first need the notion of a *deterministic and complete* program. The definition is somewhat different from the standard definition for finite automata, since it takes the semantic meaning of constraints into account.

Intuitively, in a deterministic and complete program, every concrete run has exactly one trace that induces it.

**Definition 5.** *A program over alphabet $\alpha$ is* deterministic and complete *if for every state $q$ and for every action $a \in \alpha$ the following hold:*

*1. There is exactly one state $q'$ such that $(q, a, q')$ is in $\delta$.*[5]

[5] in our examples we sometimes omit the actions that lead to a rejecting sink for the sake of clarity.

2. *If* $(q, c_1, q')$ *and* $(q, c_2, q'')$ *are in* $\delta$ *for constraints* $c_1, c_2 \in \mathcal{C}$ *and* $q' \neq q''$*, then* $c_1 \wedge c_2 \equiv false$.
3. *Let* $C_q$ *be the set of all constraints on transitions leaving q. Then* $(\bigvee_{c \in C_q} c) \equiv true$.

A *property* is a deterministic and complete program with no assignment actions.

A trace is accepted by a property $P$ if it reaches a state in $F$, the set of accepting states of $P$. Otherwise, it reaches a state in $Q \setminus F$, and is rejected by $P$.

Next, we define the satisfaction relation $\models$ between a program and a property. Intuitively, a program $M$ satisfies a property $P$ (denoted $M \models P$) if all runs induced by accepted traces of $M$ reach an accepting state in $P$.

A property $P$ specifies the behavior of a program $M$ by referring to communication actions of $M$ and imposing constraints over the variables of $M$. Thus, the set of variables of $P$ is identical to that of $M$. Let $\mathcal{G}$ be the set of communication actions of $M$. Then, $\alpha P$ includes a subset of $\mathcal{G}$ as well as constraints over the variables of $M$. The *interface* of $M$ and $P$, which consists of the communication actions that occur in $P$, is defined as $\alpha I = \mathcal{G} \cap \alpha P$.

In order to capture the satisfaction relation between $M$ and $P$, we define a *conjunctive composition* between $M$ and $P$, denoted $M \times P$. In conjunctive composition, the two components synchronize on their common communication actions when both read or both write through the same communication channel. They interleave on constraints and on actions of $\alpha M$ that are not in $\alpha P$.

**Definition 6.** *Let* $M = \langle Q_M, X_M, \alpha M, \delta_M, q_0^M, F_M \rangle$ *be a program and* $P = \langle Q_P, X_P, \alpha P, \delta_P, q_0^P, F_P \rangle$ *be a property, where* $X_M = X_P$*. The* conjunctive composition *of M and P is* $M \times P = \langle Q, X, \alpha, \delta, q_0, F \rangle$*, where:*

1. $Q = Q_M \times Q_P$*. The initial state is* $q_0 = (q_0^M, q_0^P)$.
2. $X = X_M = X_P$.
3. $\alpha = \{ g!x, g?x, (g?x, g!y), (g!x, g?y) \mid g * x, (g * x, g * y) \in \alpha I \} \cup ((\alpha M \cup \alpha P) \setminus \alpha I))$[6]*. That is, the alphabet includes communication actions on channels common to M and P. It also includes individual actions of M and P.*
4. $\delta$ *is defined as follows.*
   (a) *For* $a = (g * x, g * y) \in \alpha I$*, or* $a = g * x \in \alpha I$*:* $\delta((q_1, q_2), a) = (\delta_M(q_1, a), \delta_P(q_2, a))$.
   (b) *For* $a \in \alpha M \setminus \alpha I$*:* $\delta((q_1, q_2), a) = (\delta_M(q_1, a), q_2)$.
   (c) *For* $a \in \alpha P \setminus \alpha I$*:* $\delta((q_1, q_2), a) = (q_1, \delta_P(q_2, a))$.
   *That is, on actions that are not common communication actions to M and P, the two components interleave.*
5. $F = F_M \times B_P$*, where* $B_P = Q_P \setminus F_P$.

Note that accepted traces in $M \times P$ are those that are accepted in $M$ and rejected in $P$. Such traces are called *error traces* and their corresponding runs are called *error runs*. Intuitively, an error run is a run along $M$ which violates the properties modeled by $P$. Such a run either fails to synchronize on the communication actions, or reaches a point in the computation in which its assignments, coming from $M$, violate some constraint described by $P$. These runs are manifested in the traces that are accepted in $M$ but are composed with matching traces that are rejected in $P$. We can now formally define when a program satisfies a property.

**Definition 7.** *For a program M and a property P, we define* $M \models P$ *iff* $M \times P$ *contains no feasible accepted traces.*

[6] Note that communication actions of the form $(g * x, g * y)$ can only appear if $M$ is a parallel composition of two programs.

Thus, a feasible error trace in $M \times P$ is an evidence to $M \not\models P$, since it indicates the existence of a run that violates $P$.

*Example 2.* Consider the program $M$ of Figure 3 and the property $P$ of Figure 2. As we discussed in Section 1, $M \nvDash P$. The trace $t = \langle read?x_{pw}, 999 < x_{pw}, (enc!x_{pw}, enc?y_{pw}), x_{pw} == y_{pw}, y_{pw} := 2 \cdot y_{pw}, (getEnc?x_{pw2}, getEnc!y_{pw}), x_{pw2} == y_{pw}, x_{pw}! = x_{pw2}, y_{pw} \geq 2^{64}\rangle$ is a feasible error trace in $M \times P$ proving that an overflow is possible.

## 4 The Assume-Guarantee-Repair (AGR) Framework

In this section we discuss our Assume-Guarantee-Repair (AGR) framework for communicating programs. The framework consists of a learning-based Assume-Guarantee algorithm, called $AG_{L^*}$, and a REPAIR procedure, which are tightly joined.

Let $M_1$ and $M_2$ be two programs, and let $P$ be a property. The classical Assume-Guarantee (AG) proof rule [26] assures that if we find an assumption $A$ (in our case, a communicating program) such that $M_1||A \models P$ and $M_2 \models A$ both hold, then $M_1||M_2 \models P$ holds as well. For LTSs [9], the AG-rule is guaranteed to either prove correctness or return a real (non-spurious) counterexample. The work in [9] relies on the $L^*$ algorithm [5] for learning an assumption $A$ for the AG-rule. In particular, $L^*$ aims at learning $A_w$, the weakest assumption for which $M_1||A_w \models P$ holds. A crucial point of this method is the fact that $A_w$ is *regular* [15], and thus can be learned by $L^*$.

**Lemma 1.** *For infinite-state communicating programs, the weakest assumption $A_w$ is not always regular.*

*Example 3.* Consider the programs $M_1, M_2$ and the property $P$ of Figure 4. The weakest assumption with which $M_1$ satisfies $P$ should contain exactly all traces (over the alphabet of $M_2$) that contain equally many actions of the form $x := x + 1$ and $y := y + 1$. This set of traces is not regular, and therefore cannot be learned by $L^*$.

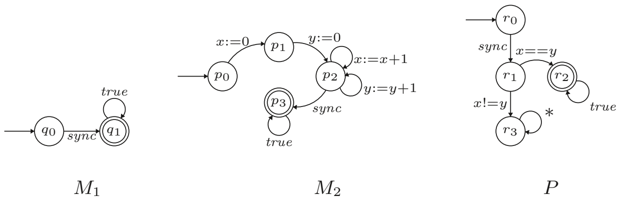

Fig. 4: A system for which the weakest assumption is not regular

To cope with this difficulty, we change the target of learning. Instead of learning the (possibly) non-regular language of $A_w$, we learn $\mathcal{T}(M_2)$, the set of accepted traces of $M_2$. This language is guaranteed to be regular, as it is represented by the automaton $M_2$.

Note that in case that $M_1||M_2 \models P$, repair is never needed, and $M_2$ is a valid assumption. In the worst case, the procedure halts once it has learned $M_2$. In particular, in case there are no error traces, termination of our algorithm is guaranteed. If $M_1||M_2 \nvDash P$ then there does not exist a matching assumption, and attempting to learn $M_2$ will reveal this. Therefore, using $\mathcal{T}(M_2)$ as a learning goal matches the AG rule.

The nature of $AG_{L^*}$ is such that the assumptions it learns before it reaches $M_2$ may contain the traces of $M_2$ and more, but still be represented by a smaller automaton. Therefore, similarly to [9], $AG_{L^*}$ often terminates with an assumption $A$ that is much smaller than $M_2$. Indeed, our tool often produces very small assumptions (see Section 5).

As mentioned before, not only that we determine whether $M_1||M_2 \models P$, but we also repair the program in case it violates the specification. When $M_1||M_2 \not\models P$, the $AG_{L^*}$ algorithm returns an error trace $t$ as a witness for the violation. In this case, we initiate the REPAIR procedure, which eliminates $t$ from $M_2$. REPAIR applies abduction in order to learn a new constraint which, when added to $t$, creates an infeasible trace.[7] The new constraint enriches the alphabet in a way which may make similar traces infeasible as well. We elaborate on our use of abduction in Section 4.2. The removal of $t$ and the addition of the new constraint result in a new goal $M_2'$ for $AG_{L^*}$ to learn. We now return to $AG_{L^*}$ to search for a new assumption $A'$ that allows to verify $M_1||M_2' \models P$.

An important feature of our AGR algorithm is its *incrementality*. When learning an assumption $A'$ for $M_2'$ we can use the membership queries previously asked for $M_2$, since the answer for them has not been changed. In the full version [1] we prove that the difference between the languages of $M_2$ and $M_2'$ lies in words (traces) whose membership has not yet been queried on $M_2$. This allows the learning of $M_2'$ to start from the point where the previous learning has left off, resulting in a more efficient algorithm.

As opposed to the case where $M_1||M_2 \models P$, we cannot guarantee the termination of the repair process in case $M_1||M_2 \not\models P$. This, since we are only guaranteed to remove one (bad) trace and add one (infeasible) trace in every AGR REPAIR iteration (although in practice, every iteration may remove a larger set of traces). Thus, we may never converge to a repaired system. Nevertheless, in case of property violation, our algorithm always finds an error trace, thus a progress towards a "less erroneous" program is guaranteed.

It should be noted that the $AG_{L^*}$ part of our AGR algorithm deviates from the AG-rule of [9] in two important ways. First, since the goal of our learning is $M_2$ rather than $A_w$, our membership queries are different in type and order. Second, in order to identify real error traces and send them to REPAIR as early as possible, we add additional queries to the membership phase that reveal such traces. We then send them to REPAIR without ever passing through equivalence queries, which improves the overall efficiency. Indeed, our experiments include several cases in which all repairs were invoked from the membership phase. In these cases, AGR ran an equivalence query only when it has already successfully repaired $M_2$, and terminated.

### 4.1 The Assume-Guarantee-Repair (AGR) Algorithm

We now describe our AGR algorithm in more detail (see Algorithm 1). Figure 5 describes the flow of the algorithm. AGR comprises two main parts, namely $AG_{L^*}$ and REPAIR.

The input to AGR are the components $M_1$ and $M_2$, and the property $P$. While $M_1$ and $P$ stay unchanged during AGR, $M_2$ keeps being updated as long as the algorithm recognizes that it needs repair (we can guarantee termination in certain cases, as we discuss in Section 4.4).

The algorithm works in iterations, where in every iteration the next updated $M_2^i$ is calculated, starting with iteration $i = 0$, where $M_2^0 = M_2$. An iteration starts with the membership phase in line 2, and ends either when $AG_{L^*}$ successfully terminates (line 16) or when procedure REPAIR is called (lines 7 and 24). When a new system $M_2^i$ is constructed, $AG_{L^*}$ does not start from scratch. The information that has been used in previous iterations is still valid for $M_2^i$. The new iteration is given additional new trace(s) that have been added or removed from the previous $M_2^i$ (lines 9,11,20, 27).

$AG_{L^*}$ consists of two phases: membership, and equivalence.

The membership phase (lines 2-11) consists of a loop in which the learner constructs the next assumption $A_j^i$ according to answers it gets from the teacher on a sequence of membership queries on various

[7] There are also cases in which we do not use abduction, as discussed in Section 4.3

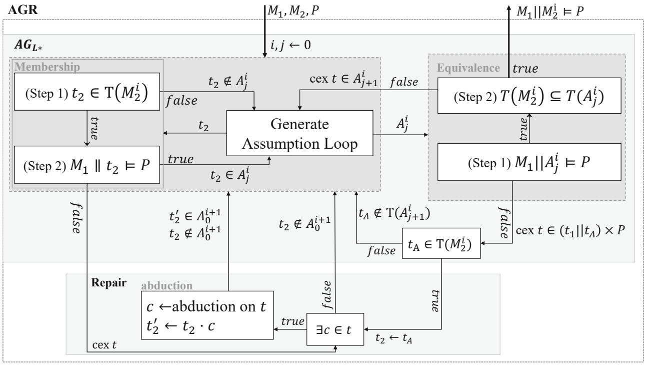

Fig. 5: The flow of AGR

traces. These queries are answered in accordance with traces we allow in $A_j^i$: traces in $M_2^i$ that in parallel with $M_1$ satisfy $P$. If a trace $t \in \mathcal{T}(M_2^i)$ in parallel with $M_1$ does not satisfy $P$, then $t$ is a bad behavior of $M_2$. Therefore, if such a $t$ is found during the membership phase, REPAIR is invoked.

Once the learner reaches a stable assumption $A_j^i$, it passes it to the equivalence phase (lines 12-27). $A_j^i$ is a suitable assumption if both $M_1||A_j^i \models P$ and $\mathcal{T}(M_2^i) \subseteq \mathcal{T}(A_j^i)$ hold. In this case, AGR terminates and returns $M_2^i$ as a successful repair of $M_2$. If $M_1||A_j^i \not\models P$, then a counterexample $t$ is returned, that is composed of bad traces in $M_1, A_j^i$, and $P$. If the bad trace $t_2$, the restriction of $t$ to the alphabet of $A_j^i$, is also in $M_2^i$, then $t_2$ is a bad behavior of $M_2^i$, and here too the REPAIR phase is invoked. Otherwise, AGR returns to the membership phase with $t_2$ as a trace that should not be in $A_j^i$, and continues to learn $A_{j+1}^i$.

As we have described, REPAIR is called when a bad trace $t$ is found in $(M_1||M_2^i) \times\ P$ and should be removed. If $t$ contains no constraints then its sequence of actions is illegal and its subtrace $t_2$ from $M_2^i$ should be removed from $M_2^i$. In this case, REPAIR returns to $AG_{L^*}$ with a new learning goal $M_2^{i+1}$ such that $\mathcal{T}(M_2^{i+1}) \subseteq \mathcal{T}(M_2^i) \setminus \{t_2\}$, along with the answer "no" to the membership query on $t_2$. In 4.3 we discuss different methods for removing $t_2$ from $M_2^i$.

The more interesting case is when $t$ contains constraints. In this case, we not only remove the matching $t_2$ from $M_2^i$, but we also add a new constraint $c$ to the alphabet of $M_2^{i+1}$, which causes $t_2$ to be infeasible. This way we eliminate $t_2$, and may also eliminate a family of bad traces that violate the property in the same manner. We deduce $c$ using abduction, see Section 4.2. As before, REPAIR returns to $AG_{L^*}$ with a new goal to be learned, but now also with an extended alphabet. The membership phase is then provided with two new answers to the membership query: $t_2$ that should *not* be included in the new assumption, and $(t_2 \cdot c)$ that should be included.

**Incremental learning** One of the advantages of AGR is that it is *incremental*, in the sense that membership answers from previous iterations remain unchanged for the repaired system. Indeed, since this is the first time that $AG_{L^*}$ queries $t_2$, we can return to $AG_{L^*}$ with the answer $t_2 \notin \mathcal{T}(M_2^{i+1})$, without contradicting any previous queries. In addition, $t_2'$ obtained by abduction is a new word (over a new alphabet), which also was not queried earlier. Therefore, we can incrementally add $t_2$ and $t_2'$ as answers from the teacher, and continue to use answers from previous queries on all other traces.

**Algorithm 1** AGR

1: **function** $AG_{L^*}$
2: //Membership Queries
3: Let $t_2 \in (\alpha M_2^i)^*$.
4: **if** $t_2 \in \mathcal{T}(M_2^i)$ **then**
5: **if** $M_1||t_2 \nvDash P$ **then**
6: Let $t \in (M_1||t_2) \times P$ be an error trace. $\triangleright$ $t$ is a cex proving $M_1||M_2^i \nvDash P$
7: REPAIR($M_2^i, t$)
8: **else** $\triangleright$ $M_1||t_2 \vDash P$
9: Return to $AG_{L^*}$ in Line 2 with $t_2 \in \mathcal{T}(A_j^i)$.
10: **else** $\triangleright$ $t_2 \notin \mathcal{T}(M_2^i)$
11: Return to $AG_{L^*}$ in Line 2 with $t_2 \notin \mathcal{T}(A_j^i)$.
12: //Equivalence Queries
13: Let $A_j^i$ be the candidate assumption generated by the learner.
14: **if** $M_1||A_j^i \vDash P$ **then**
15: **if** $\mathcal{T}(M_2^i) \subseteq \mathcal{T}(A_j^i)$ **then**
16: Terminate and return $M_1||M_2^i \vDash P$.
17: **else**
18: Let $t_2 \in \mathcal{T}(M_2^i) \setminus \mathcal{T}(A_j^i)$.
19: Set $j := j + 1$
20: Return to $AG_{L^*}$ in Line 2 with $t_2 \in \mathcal{T}(A_j^i)$.
21: **else** $\triangleright$ $M_1||A_j^i \nvDash P$
22: let $t \in (M_1||A_j^i) \times P$ be an error trace, and denote $t = (t_1||t_A) \times t_P$.
23: **if** $t_A \in \mathcal{T}(M_2^i)$ **then**
24: REPAIR($M_2^i, t_A$) $\triangleright$ $t_A$ is a cex proving $M_1||M_2^i \nvDash P$
25: **else**
26: Set $j := j + 1$.
27: Return to $AG_{L^*}$ in Line 2 with $t_A \notin \mathcal{T}(A_j^i)$.
28: **function** REPAIR($M_2^i, t$)
29: Let $t_1 \in M_1, t_2 \in M_2^i, t_p \in P$ such that $t = (t_1||t_2) \times t_p$.
30: **if** $t$ does not contain constraints **then**
31: Return to $AG_{L^*}$ in Line 2 with $M_2^{i+1}$ such that $\mathcal{T}(M_2^{i+1}) = \mathcal{T}(M_2^i) \setminus \{t_2\}$ and $t_2 \notin \mathcal{T}(A_0^{i+1})$.
32: **else** $\triangleright$ $t$ contains constraints
33: Use *abduction* to eliminate $t$.
34: Let $c$ be the new constraint learned during abduction.
35: Update $\alpha M_2^{i+1} = \alpha M_2^i \cup \{c\}$.
36: Let $t_2' = t_2 \cdot c$ be the output of the abduction.
37: Return to $AG_{L^*}$ in Line 2 with $M_2^{i+1}$ such that $\mathcal{T}(M_2^{i+1}) = (\mathcal{T}(M_2^i) \setminus \{t_2\}) \cup \{t_2'\}$,
38: and $t_2 \notin \mathcal{T}(A_0^{i+1}), t_2' \in \mathcal{T}(A_0^{i+1})$

### 4.2 Repair by Abduction

We now describe the repair we apply to $M_2^i$, in case the error trace $t$ contains constraints (see Algorithm 1, line 32). Error traces with no constraints are removed from $M_2^i$ syntactically (line 31), while in abduction we *semantically* eliminate $t$ by making it infeasible. The new constraints are then added to the alphabet of $M_2^{i+1}$ in a way that may eliminate additional error traces. Note that even-though we add new alphabet letters to $M_2$, we do not add new *feasible traces*, since the constraints added by abduction can only restrict the behavior of $M_2$, making more traces infeasible. Therefore, we do not add counterexamples to $M_2$.

The process of inferring new constraints from known facts about the program is called *abduction* [11]. We now describe how we apply it. Given a trace $t$, let $\varphi_t$ be the first-order formula (a conjunction of constraints), which constitutes the SSA representation of $t$ [4]. In order to make $t$ infeasible, we look for a formula $\psi$ such that $\psi \wedge \varphi_t \rightarrow \mathit{false}$[8].

Note that $t \in \mathcal{T}(M_1||M_2^i) \times P$, and so it includes variables both from $X_1$, the set of variables of $M_1$, and from $X_2$, the set of variables of $M_2^i$. Since we wish to repair $M_2^i$, the learned $\psi$ is over the variables in $X_2$ only.

The formula $\psi \wedge \varphi_t \rightarrow \mathit{false}$ is equivalent to $\psi \rightarrow (\varphi_t \rightarrow \mathit{false})$. Thus, $\psi = \forall x \in X_1(\varphi_t \rightarrow \mathit{false}) \equiv \forall x \in X_1(\neg\varphi_t)$, is such a desired constraint: $\psi$ makes $t$ infeasible and is defined only over $X_2$. We now use quantifier elimination [28] to produce a quantifier-free formula over $X_2$. Computing $\psi$ is similar to the abduction suggested in [11], but the focus here is on finding a formula over $X_2$ rather than over any minimal set of variables. We use Z3 [10] to apply quantifier elimination and to generate the new constraint. After generating $\psi(X_2)$, we add it to the alphabet of $M_2^{i+1}$ (line 35 of Algorithm 1). In addition, we produce a new trace $t_2' = t_2 \cdot \psi(X_2)$. The trace $t_2'$ is returned as the output of the abduction.

*Example 4.* Recall the error trace $t = \langle \mathit{read}?x_{pw}, 999 < x_{pw}, (\mathit{enc}!x_{pw}, \mathit{enc}?y_{pw}), x_{pw} == y_{pw}, y_{pw} := 2 \cdot y_{pw}, (\mathit{getEnc}?x_{pw2}, \mathit{getEnc}!y_{pw}), x_{pw2} == y_{pw}, x_{pw}! = x_{pw2}, y_{pw} \geq 2^{64}\rangle$ of Example 2. From $t$ we create the formula $\varphi_t = (999 < x_{pw}) \wedge (y_{pw} = x_{pw}) \wedge (y'_{pw} = 2 \cdot y_{pw}) \wedge (x_{pw2} = y'_{pw}) \wedge (x_{pw} \neq x_{pw2}) \wedge (y'_{pw} \geq 2^{64})$. We then apply quantifier elimination and simplification on the formula $\forall y_{pw} \forall y'_{pw}(\neg\varphi_t)$ and get the new constraint $x_{pw} < 2^{63}$.

**Lemma 2.** *Let $t = (t_1||t_2) \times t_P$. If $t_2$ is infeasible, then $t$ is infeasible as well.*

This is due to the fact that $t_P$ can only restrict the behaviors of $t_1$ and $t_2$, thus if $t_2$ is infeasible, $t$ cannot be made feasible. See the full version of the paper [1] for a formal proof. Therefore, by making $t_2$ infeasible, we eliminate the error trace $t$.

We now want to build a repaired component $M_2^{i+1}$ of $M_2^i$, which includes $t_2 \cdot \psi(X_2)$ but not $t_2$. To do so, we split the state $q$ that $t_2$ reaches in $M_2^i$ into two states $q, q'$, and add a transition labeled $\psi(X_2)$ from $q$ to $q'$, where only $q'$ is now accepting[9]. Thus, we eliminated a violating trace from $M_1||M_2^i$. AGR now returns to $AG_{L^*}$ in order to learn an assumption for the repaired component $M_2^{i+1}$, which now includes $t_2'$ but not $t_2$.

### 4.3 Removal of Error Traces

Recall that the goal of REPAIR is to remove a bad trace $t$ from $M_2$ once it is found by $AG_{L^*}$. If $t$ contains constraints, we remove it using abduction. Otherwise, we can remove $t$ by constructing a system whose language is $\mathcal{T}(M_2) \setminus \{t\}$. We call this the *exact* method for repair. However, removing a single trace at a time may lead to slow convergence, and to an exponential blow-up in the size of the repaired systems. Moreover, as we have discussed, in some cases there are infinitely many such error traces, in which case AGR may never terminate.

For faster convergence, we have implemented two additional heuristics, namely *approximate* and *aggressive*. These heuristics may remove more than a single trace at a time, while keeping the size of the systems small. While "good" traces may be removed as well, the correctness of the repair is maintained, since no bad traces are added. Moreover, an error trace is likely to be in an erroneous part of the system, and in these cases our heuristics manage removing a set of error traces in a single step.
We briefly survey the three methods.

[8] Usually, in abduction, we look for $\psi$ such that $\psi \wedge \varphi_t$ is not a contradiction. In our case, however, since $\varphi_t$ is a violation of the specification, we want to infer a formula that makes $\varphi_t$ unsatisfiable.

[9] Note that $q$ is an accepting state in $M_2^i$ since $t \in \mathcal{T}(M_2^i)$.

- *Exact.* To eliminate only $t$ from $M_2$, we construct a program (an automaton) $A_t$ that accepts only $t$, and complement it to construct $A'_t$ that accepts all traces except for $t$. Finally, we intersect $A'_t$ with $M_2$.
- *Approximate.* Similarly to our repair via abduction in Section 4.2, we prevent the last transition that $t$ takes from reaching an accepting state. Let $q$ be the state that $t$ reaches. We mark $q$ as non-accepting, and add an accepting state $q'$, to which all in-going transitions to $q$ are diverted, except for the last transition on $t$. This way, some traces that lead to $q$ are preserved by reaching $q'$ instead, and the traces that share the last transition of $t$ are eliminated along with $t$. As we have argued, these transitions may also be erroneous.
- *Aggressive.* In this simple method, we remove $q$, the state that $t$ reaches, from the set of accepting states. This way we eliminate $t$ along with all other traces that lead to $q$. In case that every accepting state is reached by some error trace, this repair might result in an empty language, creating a trivial repair. However, our experiments show that in most cases, this method quickly leads to a non-trivial repair.

### 4.4 Correctness and Termination

For this discussion, we assume a sound and complete teacher who can answer the membership and equivalence queries in $AG_{L^*}$, which require verifying communicating programs and properties with first-order constraints.

As we have discussed earlier, AGR is not guaranteed to terminate, and there are cases where the REPAIR stage may be called infinitely many times. However, in case that no repair is needed, or if a repaired system is obtained after finitely many calls to REPAIR, then AGR is guaranteed to terminate with a correct answer.

To see why, consider a repaired system $M_2^i$ for which $M_1||M_2^i \models P$. Since the goal of $AG_{L^*}$ is to syntactically learn $M_2^i$, which is regular, this stage will terminate at the latest when $AG_{L^*}$ learns exactly $M_2^i$ (it may terminate sooner if a smaller appropriate assumption is found). Notice that, in particular, if $M_1||M_2 \models P$, then AGR terminates with a correct answer in the first iteration of the verify-repair loop.

REPAIR is only invoked when a (real) error trace $t$ is found in $M_2^i$, in which case a new system $M_2^{i+1}$, that does not include $t$, is produced by REPAIR. If $M_1||M_2^i \not\models P$, then an error trace is guaranteed to be found by $AG_{L^*}$ either in the membership or equivalence phase. Therefore, also in case that $M_2^i$ violates $P$, the iteration is guaranteed to terminate. To conclude, we have the following.

**Theorem 1.**
- *An iteration $i$ of AGR ends with an error trace $t$ iff $M_1||M_2^i \not\models P$, where $M_2^i$ is the repaired system at iteration $i$.*
- *If, after finitely many iterations, a repaired program $M'_2$ is such that $M_1||M'_2 \models P$, then AGR terminates with a correct answer.*

We have shown that every iteration of AGR is guaranteed to terminate with a correct answer. The detailed correctness proofs are in the full version of this paper [1].

In particular, since every iteration of AGR finds and removes an error trace $t$, and no new erroneous traces are introduced in the updated system, then in case that $M_2$ has finitely many error traces, AGR is guaranteed to terminate with a correctly repaired system.

## 5 Experimental Results and Conclusions

We implemented our AGR framework in Java, integrating $L^*$ implementation from the LTSA tool [20]. We used Z3 [10] as the teacher for the satisfaction queries in $AG_{L^*}$, and for abduction in REPAIR.

Table 1 displays some results of running AGR on various examples, varying in their sizes, types of errors – semantic and syntactic – and their amount. Additional results are in the full version of this paper [1], and the full examples are available on [2]. The *iterations* column indicates the number of iterations of the verify-repair loop, until a repaired $M_2$ is achieved. Examples with no errors were verified in the first iteration, and are indicated by *verification*. We tested the three repair methods described in Section 4.3 for counterexamples without constraints, and used abduction when needed. Figure 6 presents comparisons between the three methods in terms of run-time and the size of the repair and assumptions (note that the graphs are given in logarithmic scale).

Table 1: AGR algorithm results on various examples

| **Example** | $M_1$ **Size** | $M_2$ **Size** | $P$ **Size** | **Time (sec.)** | $A$ **size** | **Repair Size** | **Repair Method** | **#Iterations** |
|---|---|---|---|---|---|---|---|---|
| #4 | 64 | 64 | 3 | 95 | 7 | verification | | |
| #6 | 2 | 27 | 2 | 0.106 | 5 | 27 | aggress. | 2 |
| | | | | 0.126 | 6 | 28 | approx. | 2 |
| | | | | 0.132 | 8 | 81 | exact | 2 |
| #7 | 2 | 81 | 2 | 0.13 | 6 | 81 | aggress. | 2 |
| | | | | 0.138 | 7 | 82 | approx. | 2 |
| | | | | 0.165 | 9 | 243 | exact | 2 |
| #8 | 2 | 243 | 2 | 0.15 | 8 | 243 | aggress. | 2 |
| | | | | 0.17 | 8 | 244 | approx. | 2 |
| | | | | 0.223 | 10 | 729 | exact | 2 |
| #11 | 5 | 256 | 6 | 4.88 | 92 | verification | | |
| #14 | 5 | 256 | 6 | 4.44 | 109 | verification | | |
| #15 | 3 | 16 | 5 | 0.69 | 12 | 16 | aggress. | 5 |
| | | | | 0.28 | 13 | 18 | approx. | 3 |
| | | | | 4.27 | 44 | 864 | exact | 5 |
| #16 | 4 | 256 | 8 | 6.63 | 113 | 256 | aggress. | 2 |
| | | | | 5.94 | 113 | 257 | approx. | 2 |
| | | | | 12.87 | 155 | 1280 | exact | 2 |
| #19 | 3 | 16 | 5 | 1.07 | 18 | 18 | aggress. | 3 |
| | | | | 1.12 | 18 | 18 | approx. | 3 |
| | | | | 1.26 | 18 | 18 | exact | 3 |
| #22 | 2 | 4 | 2 | 0.09 | 1 | 4 (trivial) | aggress. | 4 |
| | | | | 0.21 | 6 | 8 | approx. | 5 |
| | | | | timeout | | | exact | timeout |

Most of our examples model multi-client-server communication protocols, with varying sizes. Our tool managed repairing all these examples when needed.

As can be seen in Table 1, our tool successfully generates assumptions that are significantly smaller than the repaired and the original $M_2$.

For the examples that needed repair, in most cases our tool needed 2-5 iterations of verify-repair in order to successfully construct a repaired component. Interestingly, in example $\#15$ the *aggressive* method converged slower than the *approximate* method. This is due to the structure of $M_2$, in which different error traces lead to different states. Marking these states as non-accepting removed each trace separately. However, some of these traces have a common transition, and preventing this transition from reaching an accepting state, as done in the *approximate* method, managed removing several error traces in a single repair. This example also includes repairs by abduction (as do examples $\#16$, $\#18$ and $\#19$).

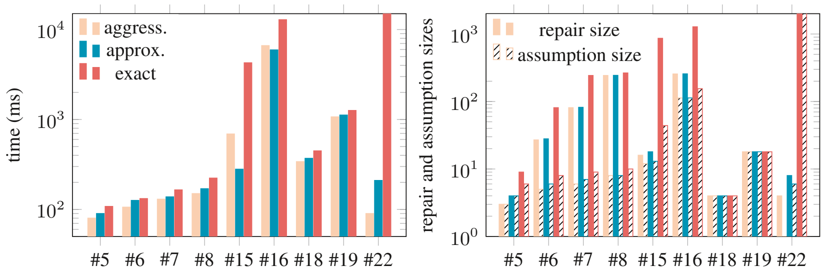

Fig. 6: Comparing repair methods: time and repair size (logarithmic scale).

Example $\#22$ models a simple structure in which, due to a loop in $M_2$, the same alphabet sequence can generate infinitely many error traces. The *exact* repair method timed out, since it attempted removing one error trace at a time. On the other hand, the *aggressive* method removed all accepting states, creating an empty program – a trivial (yet valid) repair. However, the *approximate* method created a valid, non-trivial repair.

**Conclusion** AGR offers a new take on the learning-based approach to assume-guarantee verification, and manages coping with complex properties and repairing infinite-state programs. Our experimental results show that using existing semantic tools, AGR produces very succinct proofs, and quickly and efficiently repairs flawed communicating programs.
